# Assessment of epilepsy using noninvasive visual psychophysics tests of surround suppression

**DOI:** 10.14814/phy2.13079

**Published:** 2017-03-08

**Authors:** Partow Yazdani, Jenny C.A. Read, Roger G. Whittaker, Andrew J. Trevelyan

**Affiliations:** ^1^ Institute of NeuroscienceMedical SchoolNewcastle UniversityFramlington PlaceNewcastle upon TyneUnited Kingdom

**Keywords:** Epilepsy, surround suppression, visual psychophysics

## Abstract

Powerful endogenous inhibitory mechanisms are thought to restrict the spread of epileptic discharges in cortical networks. Similar inhibitory mechanisms also influence physiological processing. We reasoned, therefore, that useful information about the quality of inhibitory restraint in individuals with epilepsy may be gleaned from psychophysical assays of these physiological processes. We derived a psychophysical measure of cortical inhibition, the motion surround suppression index (SSI), in 54 patients with epilepsy and 146 control subjects. Multivariate regression analyses showed that SSI was predicted strongly by age and seizure type, but not by seizure frequency. Specifically, we found that patients with exclusively focal epilepsy, and no history of generalization, showed significantly stronger cortical inhibition as measured by the SSI compared to all other groups, including controls. In contrast, patients with focal seizures evolving into generalized seizures, and patients with generalized genetic epilepsy, showed similar levels of cortical inhibition to controls. The presumptive focus, when one could be identified, was rarely found in visual cortex, meaning that the relationship with the epilepsy subtype is likely to reflect some global difference in inhibition in these subjects. This is the first reported instance of raised SSI in any patient cohort, and appears to differentiate between patients with respect to the likelihood of their experiencing generalization of their seizures. These results suggest that such simple psychophysical assays may provide useful aids to clinical management, particularly at the time of diagnosis.

## Introduction

Most epileptic seizures are thought to arise from impaired interactions between excitatory and inhibitory elements in the cerebral and hippocampal cortices. A key role appears to be played by an endogenous inhibitory restraint mechanism arising from the particular arrangement of inhibitory drives onto pyramidal cells, and which serves to oppose the spread of epileptic activity (Prince and Wilder 1967; Trevelyan et al. 2006; Trevelyan and Schevon 2013). The inhibitory effects provided by the cortical interneurons (Atallah et al. 2012; Wilson et al. 2012; Pouille et al. 2013) are apparent in recordings of primary visual cortex neurons during various forms of visual suppression (Sengpiel et al. 1998). The same inhibitory networks are also believed to underlie various perceptual phenomena, collectively known as psychophysical surround suppression (Tadin et al. 2003, 2006a; Betts et al. 2009; Golomb et al. 2009; Serrano‐Pedraza et al. 2014; Tadin 2015; Yazdani et al. 2015), although this does not discount contributions from other non‐GABAergic mechanisms (Tadin 2015). One such test is based on the paradoxical finding that one's ability to perceive the direction of movement of a high‐contrast sinusoidal grating is reduced, as the stimulus size is increased (Tadin et al. 2003). This is believed to arise from surround suppression in the motion visual area, MT (Tadin et al. 2003, 2006a; Tadin 2015). This psychophysical phenomenon can be represented as a single number, the surround suppression index (SSI), derived from the ratio of the duration thresholds of the large and small stimuli. Intriguingly, the SSI decreases with age (Betts et al. 2005; Yazdani et al. 2015), and is also significantly reduced in subjects with schizophrenia (Tadin et al. 2006a; Serrano‐Pedraza et al. 2014) and depression (Golomb et al. 2009); in each case, this has been proposed to reflect deficits in cortical GABAergic inhibition. We therefore investigated whether changes in SSI are also found in people with epilepsy.

We hypothesized that patients with epilepsy would also show alterations in visual psychophysical performance, and that this may be a useful clinical indicator of seizure risk. We investigated whether the SSI correlated with clinical features such as seizure frequency and seizure type, in order to determine what, if any, prognostic value might be provided by this simple psychophysics assay. Specifically, we had hypothesized that people with epilepsy might show evidence of reduced cortical inhibition, but surprisingly, our data suggest otherwise. Contrary to our original hypothesis, we found that people with generalized epilepsy showed no difference in SSI from control groups; and patients with focal epilepsy that did not generalize, on the other hand, showed a higher SSI. This is the first identified clinical group to show higher values of this measure. We suggest that these patients have enhanced cortical inhibition, which may be a factor in their seizures being restrained to subregions of the cortex. We discuss possible clinical implications of these results.

## Materials and Methods

Experimental procedures were approved by Newcastle and North Tyneside Research Ethics Committee (reference number 09/H0906/90). Participants gave written informed consent, and were paid a nominal fee for their participation. Fifty‐four patients with epilepsy (mean age, 41.9 years; age range = 17.0–82.3 years; mean duration 16.8 years; duration range = 0–50 years; 30 male) were recruited via specialist tertiary epilepsy clinics in Newcastle upon Tyne, UK. Seizure types and presumed etiology were classified according to the recent ILAE guidelines (Berg et al. 2010). Seizure frequency was estimated from patient diaries or hospital records. Due to the well‐recognized inaccuracies of patient self‐reporting of seizures (Hoppe et al. 2007), we subdivided seizure frequency into five bins: <1/year, <1/month, <1/week, <1/day, and >1/day. A total of 146 control subjects (mean age, 36.6 years; age range = 17.3–69.1 years; 59 male) were recruited via the Newcastle University volunteer cohort. Patients filled out a questionnaire regarding concurrent health issues and current medication, and also completed an Addenbrooke's Cognitive Examination (ACE).

All subjects performed a motion discrimination task as described previously (Tadin et al. 2003; Yazdani et al. 2015). Briefly, drifting sinusoidal grating patches were presented at two different contrasts, high (peak contrast 92%) and low (2.8%), either on a desktop computer (Dell) with a CRT monitor, or a tablet computer (Samsung 700T), both running custom written Matlab software, implemented using Psychtoolbox3 (Kleiner et al. 2007). The discrimination was a simple two‐choice paradigm, with the gratings moving either left or right. The stimulus presentation duration was either shortened or lengthened depending on whether the previous response was correct or incorrect, resulting in a staircase which settled close to the duration threshold. Three staircases were run in parallel, with trials interleaved at random. The SSI was defined as the log ratio of the duration thresholds of the large and small stimuli.

There was no apparent difference in the SSI measured on the two different systems (13 subjects [9 patients, 4 controls]; SSI_system 1_ = 0.56 [range −0.16 to 1.56]; SSI_system 2_ = 0.57 [range: −0.26 to 1.23]), so all the data were pooled. Grating patches were either small (subtending 0.7° on the retina when the tablet was held at 50 cm [or 100 cm when using the desktop system], users were instructed to hold the tablet at about this distance) or large (5°), and moved either left or right at constant horizontal velocity of 2°/sec. We were able to train most patients to do these tests very easily, meaning that a dataset could be attained within 10–15 min. Three patients were unable to do the test and were excluded from the analyses. The duration of stimulus presentation was varied according to an adaptive staircase, whereby correct answers led to shorter presentations of the gratings, while incorrect answers caused the stimulus duration to be increased. Three staircase runs were interleaved randomly, so that the consequences of a correct or incorrect answer were hidden from the subject during the test. Such staircases rapidly tend toward presentations close to the threshold duration at which subjects could reliably identify the direction of movement. The actual value for the threshold duration, defined as the value where performance reached 82% (following Tadin et al. 2003), was estimated by fitting a psychometric function (Watson and Pelli 1983) to all trial durations plotted against the binary answer (right = 1; wrong = 0). A bootstrap resampling technique was used to derive 95% confidence intervals for the fitted thresholds, as described previously (Read et al. 2015). The SSI, as defined by Tadin et al. (2003), is the log ratio of the threshold durations (TD) for the large and small, high‐contrast stimuli, calculated as follows:

$$\text{SSI}=\log_{10}({\text{TD}}_{\mathrm{high}:\mathrm{large}}/\left({\text{TD}}_{\mathrm{high}:\mathrm{small}}\right)$$

Although the results for low‐contrast stimuli are not incorporated directly into this index, they provide an important control assay of whether the participants were doing the psychophysics test correctly and prove that the high‐contrast stimulus was well above their contrast threshold. Since the threshold duration for a large high‐contrast stimulus is typically longer than for a small stimulus (Tadin et al. 2003; Yazdani et al. 2015), the SSI tends to be positive, and increasingly positive values indicate stronger surround suppression.

All statistical analyses were performed using the Matlab statistical toolbox. Comparisons of two regressions were performed using ANCOVA (analysis of covariance, *aoctool* in Matlab). Multivariate linear regression used the *fitlm* tool in Matlab, treating the epilepsy subtypes as “categorical.” Model comparisons were made using the adjusted *R* ^2^ values, which takes into account the effect of adding predictors on *R* ^2^. Results were considered significant if *P* < 0.05.

## Results

**Figure 1:**
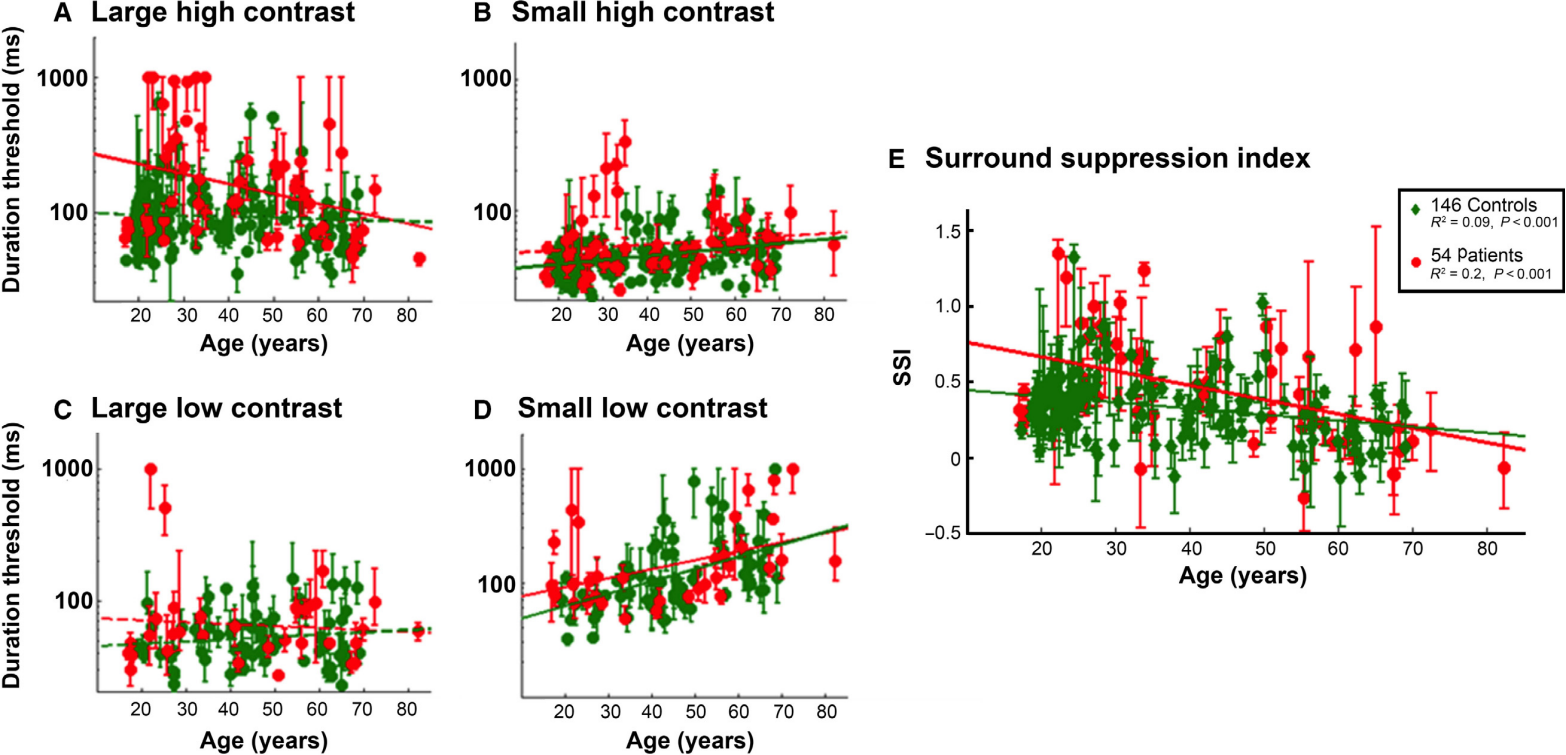
Altered psychophysical measure of surround inhibition in patients with epilepsy. (A–D) Threshold durations for discriminating the direction of movement, for large (5° field of view) and small (0.7°) gratings, at high (92%) and low contrast (2.8%), in the control and epilepsy cohorts, plotted against age of subject. The error bars for the individual data points indicate the 95% confidence interval for each, as described in our previous paper (Yazdani et al. 2015). (E) The motion surround suppression index for the control and epilepsy cohorts plotted against age of subject.

We present an analysis of the performance on a simple visual psychophysics test of 54 patients with a confirmed diagnosis of epilepsy, and 146 control subjects. Details of the individual patients are provided in Table 1. Results from a subset of the control group (36 of the 146) were published as part of a prior study (Yazdani et al. 2015); the extended dataset we show here confirm our previous reports that the SSI shows a highly significant negative correlation with age (*P* < 0.001; Fig. 1E [green diamonds]). The epilepsy cohort showed a similar, highly significant regression with age (*P* < 0.001), and furthermore, regression analysis showed that the epilepsy group was highly significantly different from the control group (*F*_1,196_ = 7.15, *P* < 0.0001), both with respect to the intercept (*P* < 0.0001) and the gradient (*P* < 0.0001) of the relationship with age. Analysis of the component tests (Fig. 1A–D) indicated that the epilepsy cohort differed only on the tests involving large high‐contrast stimuli (*F*_1,196_ = 10.08, *P* = 0.0012), which importantly is the one in which surround inhibition is likely to be manifest (Barlow and Mollon 1982; Sengpiel et al. 1998) (other tests: small high contrast, *F*_1,196_ = 1.33, *P* = 0.250; small low contrast, *F*_1,102_ = 0.02, *P* = 0.875; large low contrast, *F*_1,104_ = 3.51, *P* = 0.064; all nonsignificant). These results suggest that grouped together, the epilepsy patients have a higher SSI, suggestive of enhanced cortical inhibitory mechanism, when compared with age‐matched control subjects.

**Table 1:** Patient data

	Index	Gender	Age (years)	Age at onset (years)	Duration of epilepsy (years)	Presumed location	Seizure frequency	Antiepileptic drugs	SSI
Focal+	EP1	M	49	Not known	Not known	Temporal	3	VPA/PHT/CLB/PGB	0.09
	EP5	M	35	34	1	Frontal	2	CBZ	0.28
	EP6	F	26	1	25	Temporal	2	VAL/LTG/PGB/CLB	0.34
	EP13	M	61	27	34	Unknown	3	LTG	0.10
	EP14	F	27	26	1	Temporal	1	None	0.40
	EP18	F	55	41	14	Temporal	2	None	0.19
	EP19	M	33	3	30	Possible frontal	4	VPA/CLB/PER/PHT	−0.08
	EP24	F	22	7	15	Occipital (L)	3	TPM/ZNS	0.19
	EP27	M	68	58	10	Unknown	1	LTG	0.05
	EP30	F	58	11	47	Unknown	3	LEV/PER	0.21
	EP31	F	57	28	29	Temporal	4	LTG/PGB	0.24
	EP33	M	82	51	31	Temporal	3	LTG	−0.07
	EP36	M	59	47	12	Parietal	2	PHT/LTG/LEV/MDZ	0.10
	EP49	F	33	4	29	Temporal (L)	4	OXC	0.30
	EP51	M	30	17	13	Unknown	2	LTG/TPM	0.75
	EP53	M	33	18	15	Frontal	3	VPA/LEV	0.65
	EP56	M	68	64	4	Temporal	2	LEV	−0.12
	EP57	M	44	9	35	Right hemisphere	3	ZNS/LEV/VPA	0.79
Focal−	EP3	M	68	12	56	Temporal	1	CBZ/LEV/LTG	0.21
	EP4	M	70	64	6	Temporal	4	LTG	0.11
	EP7	F	67	6	61	Temporal	3	PHT/LTG/LEV	−0.11
	EP17	M	42	7	35	Temporal	3	CBZ/LEV	0.42
	EP20	F	28	11	17	Frontal (L)	5	RTG/CLB/CBZ/LEV	0.87
	EP2I	M	52	21	31	Frontotemporal (L)	3	CBZ/LEV	0.72
	EP23	F	62	13	49	Temporal	4	CBZ/ZNS	0.71
	EP25	F	43	7	36	Temporal	2	ZNS	1.01
	EP26	F	73	54	19	Unknown	1	VPA	0.19
	EP32	M	56	40	16	Temporal	4	LEV/RIG	0.66
	EP34	F	34	21	13	Temporal	4	PER/LEV/PGB	1.24
	EP35	M	22	16	6	Temporal	3	CBZ/TPM/CLB	1.34
	EP37	F	27	23	4	Temporal	4	PER	1.00
	EP38	F	25	0	25	Multifocal	4	LEV/LTG/CLB	0.88
	EP39	M	34	31	3	Temporal	5	TPM/LTG/OXC	0.69
	EP41	M	26	16	10	Temporal	5	CLB/LCM/LEV/ZNS	0.52
	EP42	F	31	6	25	Temporal	3	LTG / PGB	1.02
	EP44	M	42	14	28	Frontotemporal	4	VPA/LTG	0.50
	EP45	M	35	11	24	Frontal	4	VPA/PGB/ESL/PB	0.48
	EP46	F	31	5	26	Temporal lobe	4	PGB/LEV/CBZ/PHT/CLB	0.65
	EP47	M	50	45	5	Anterior temporal (L)	4	ZNS	0.87
	EP48	F	62	46	16	Temporal	4	CBZ/CLB/VPA	0.03
	EP50	F	26	22	4	Temporal	2	None	0.79
	EP55	M	51	36	15	Temporal	3	LEV	0.57
GGE	EP8	F	41	5	36	Generalized	5	None	0.48
	EP9	M	18	12	6	Generalized	1	VPA	0.43
	EP10	M	17	16	1	Generalized	2	VPA	0.31
	EP11	M	55	54	1	Unknown	2	VPA	0.42
	EP12	M	18	17	Only 1 seizure	Occipital	1	None	0.22
	EP15	M	22	22	0	Generalized	2	None	0.36
	EP16	M	18	17	1	Generalized	1	None	0.29
	EP22	F	29	20	9	Generalized	3	LEV	0.82
	EP28	M	51	5	46	Possible frontal	3	VPA/LEV/CBZ	0.27
	EP29	F	23	11	12	Generalized	4	ZNS	1.19
	EP40	F	22	22	0	Generalized	5	LEV	0.28
	EP43	F	55	15	40	Generalized	2	PRM/PGB	−0.26

**Frequency grading**	
<1/year	1
<1/month	2
<1/week	3
<1/day	4
>1/day	5

Refer Table 3 for the code of the drug lists. SSI, surround suppression index.

**Figure 2:**
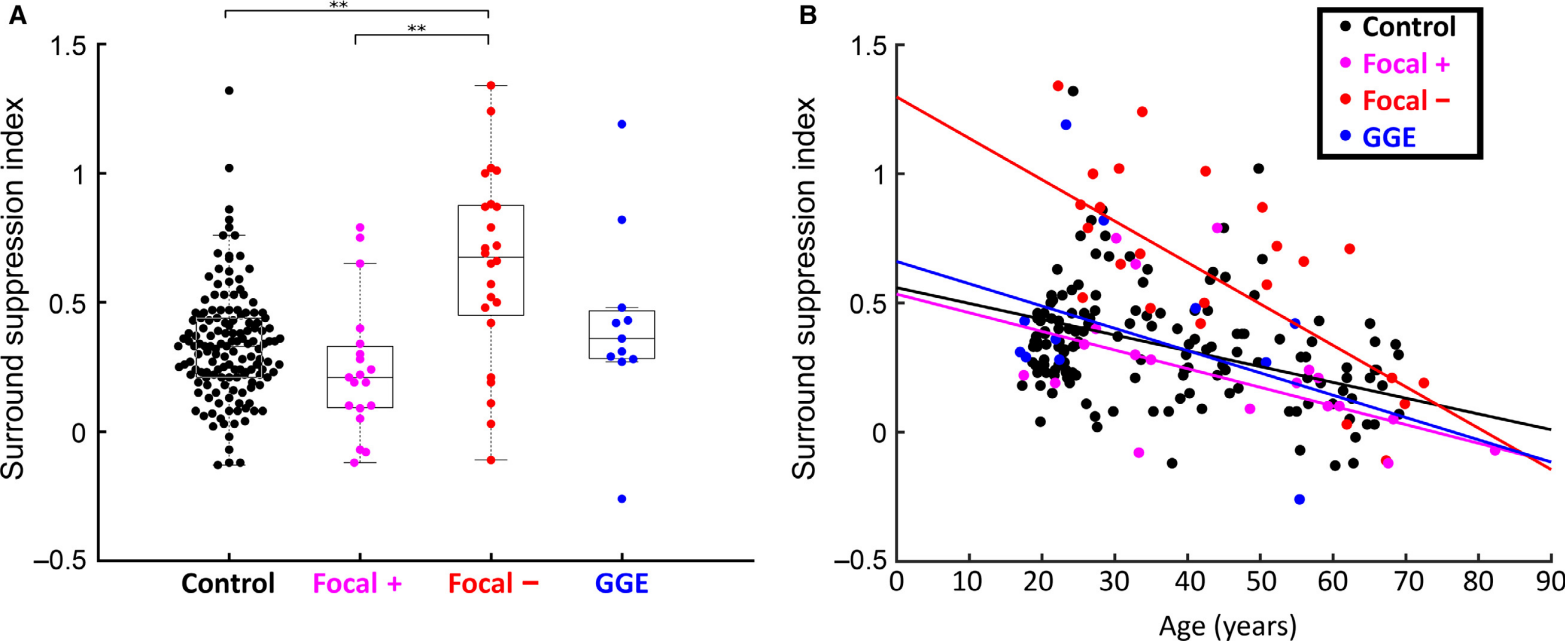
Surround suppression is altered in patients with focal nongeneralizing seizures, but is not affected by seizure frequency. (A) Box plot of SSIs for the subjects grouped by seizure type. The box limits represent the first/third quartiles, with the median indicated by the middle line and the whiskers extending to data points that are <1.5 interquartile range beyond the box. The data for the group with focal seizures without generalization (red) were highly significantly different from all other groups (**P < 0.01). (B) Regression of SSI with respect to age for the same groups of subjects. SSI, surround suppression index.

**Figure 3:**
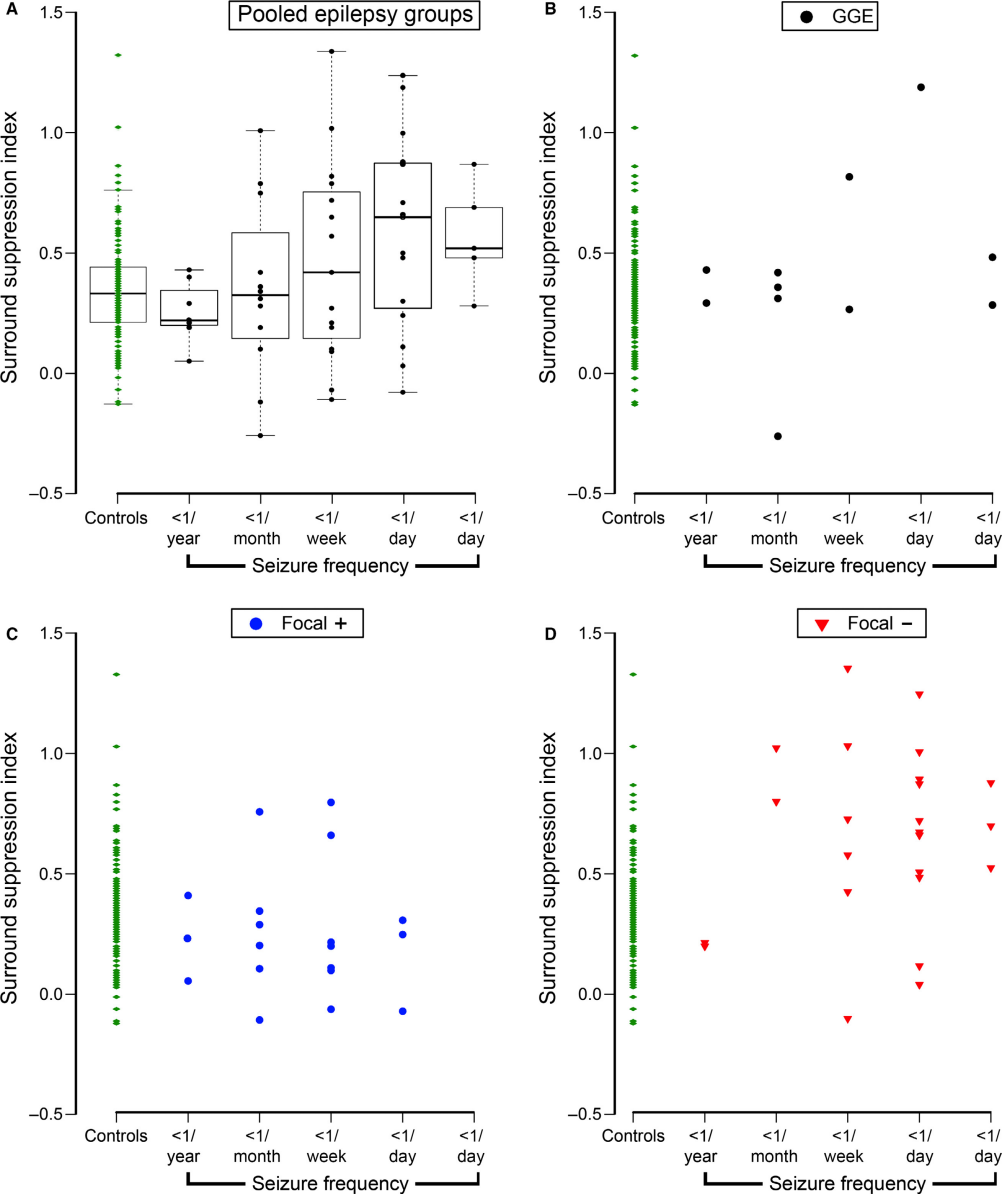
Surround suppression is not affected by seizure frequency. (A) Box plots of the SSIs with respect to frequency of seizures for the pooled epilepsy cohorts, and for each of the three subgroups of epilepsy patients, plotted separately (B–D, all nonsignificant). SSI, surround suppression index.

We next subgrouped the epilepsy cohort with respect to seizure type (Berg et al. 2010) and seizure frequency. The cohort was subclassified into three groups: those patients with focal epilepsy with a history of generalized seizures (F^+^, *n* = 19), focal epilepsy without generalizing seizures (F^−^, *n* = 24), and generalized genetic epilepsy (GGE, *n* = 11) (Fig. 2). There was no difference in performance on the Addenbrooke's Cognitive Examination between the groups (F^+^, ACE = 90.5 ± 6.2 [mean ± SD], range: 72–96; F^−^, ACE = 88.5 ± 6.3, range: 73–99; GGE, ACE = 92.0 ± 4.1, range: 85–100). Seizure frequency was binned into five groups (Fig. 3). Initial inspection of these plots suggested that, in addition to the effect of age, both seizure type and frequency might also influence the SSI. We therefore examined the relative importance of these three potential predictors (age, seizure subtype, and seizure frequency) of SSI by performing multivariate regression analyses on progressively more complex models (Table 2).

**Table 2:** Model comparisons

Models	*R* ^5^	Adjusted *R* ^2^
SSI versus Age	0.105	–
Age, epilepsy	0.195	0.183
Subtype, freq	0.170	0.144
Age, freq	0.200	0.192
Age, subtype	0.342	0.318*
Age, subtype, freq	0.342	0.315

Model parameters (age, subtype)		
	Gradient (year^−1^)	Intercept
Controls	−0.0041 ± 0.0011**	0.484 ± 0.047**
F^+^	−0.0071 ± 0.0031	0.566 ± 0.147
F^−^	−0.0164 ± 0.0031**	1.362 ± 0.141**
GGE	−0.0088 ± 0.0047	0.694 ± 0.167

Note that for the control group statistics, what is being tested is significant difference from zero, and for the other groups, it is the significant difference from the controls.

The optimal model is indicated by *, and the parameters for that model are indicated by **P << 0.001.

We first considered the subclassification into seizure types, independent of the age and seizure frequency. The distribution of SSI values differed significantly between the four groups (F^+^, F^−^, GGE, and controls; ANOVA, *F*_3,196_ = 11.66, *P* < 0.0001; Fig. 2A); *t*‐test analyses indicated that the F^−^ group was the outlier. The previously noted regression with age was apparent for each subgroup individually (Fig. 2B), although this was only significant for the two larger sample groups, F^+^ (*n* = 19, *R* ^2^ = 0.259, *P* < 0.05) and F^−^ (*n* = 24, *R* ^2^ = 0.527, *P* < 0.001), but not for GGE (*n* = 11, *R* ^2^ = 0.144, n.s.). Next, when considering both age and epilepsy diagnosis together, we found marked increases in the adjusted *R* ^2^ values when first subdividing the complete dataset (age alone, *R* ^2^ = 0105, Table 2) into controls and epilepsy subjects (adjusted *R* ^2^ = 0.183), and then further subclassifying into the F^+^, F^−^, and GGE subtypes (adjusted *R* ^2^ = 0.318). Importantly though, the age and subtype model was not further improved by adding the seizure frequency (adjusted *R* ^2^ = 0.315). This lack of effect of seizure frequency was better appreciated when this predictor was plotted for the three seizure subtypes individually (Fig. 3B–D). These plots also show that in our samples, the F^−^ patients tended toward a higher seizure frequency (the median frequency bin for GGE was “<1 month,” for F^+^ it was “<1/week,” and for F^−^ it was “<1/day”). This mismatch in the seizure frequency between the groups can explain the increase in R^2^ going from a model using just “Age” to one using “Age + Frequency” (Table 2): in this case, in which seizure subtype was ignored, the subtype acts as a hidden predictor and distorts our interpretation of the effect of frequency. The important comparison is that a model using all three predictors actually explains no more of the variance than one using just age and seizure subtype. The regression table for the three‐predictor model indicates highly significant *P* values for the control intercept and slope (*P *<< 0.001), and for the change in intercept and slope for the F^−^ group (*P* << 0.001), but for no other comparison, and notably frequency was nonsignificant (*P* = 0.632). We conclude, therefore, that only age and seizure subtypes were significant predictors of SSI.

**Figure 4:**
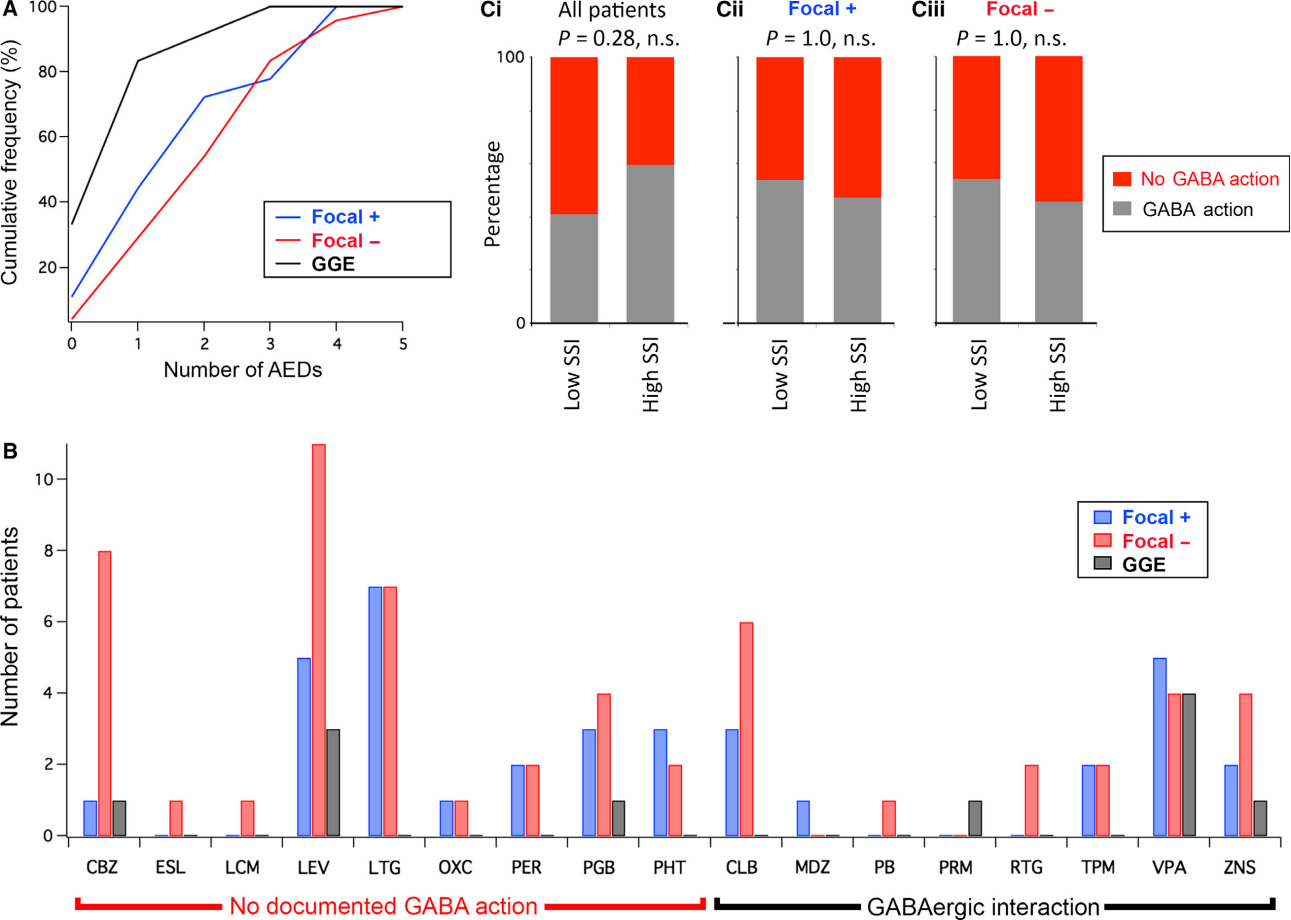
Patterns of medication for the three subgroups in the patient cohort. (A) Cumulative frequency plots of the proportions of the patients in the three groups taking different numbers of antiepileptic drugs (AEDs). (B) Histogram showing the numbers of patients in each group taking the different AEDs. The abbreviations of the drugs are given in Table 3. (C) Proportions of patients with either low SSIs or high SSIs who are on medication that either interacts with, or is considered independent of, the GABAergic system. In each case, the cohort was subdivided at the median score SSI (Ci, all patients, *n* = 27 for both low and high SSI groups; Cii, patients with generalized epilepsy [F^+^ and generalized genetic epilepsy], *n* = 15 for both groups; Ciii, patients with exclusively focal epilepsy [F^−^], *n* = 12 for both groups). SSI, surround suppression index.

One possible confounding issue was that certain antiepileptic drugs (AEDs) are known to interact with the GABAergic system, and indeed this is presumed to contribute to their clinical effect (Walker and Surges 2009). We therefore analyzed the pattern of medication of the 54 patients who participated in the study. Collectively, patients were on 17 different medications (Table 3, Fig. 4). Seven patients were recruited at the time of diagnosis and were not therefore on medication when they did the psychophysical tests; 18 patients were on monotherapy, and the rest were on multiple drugs (Fig. 4A). The GGE patient group tended to be on a lower numbers of drugs (1.00 drugs/subject), with the F^+^ and F^−^ groups taking similar numbers (1.84 and 2.33, respectively). The most commonly prescribed drugs were levetiracetam (19 patients), lamotrigine (14 patients), and sodium valproate (13 patients), but notably the pattern of drug prescriptions for the patients with generalized seizures (GGE and F^+^) and those without (F^−^) were broadly similar (Fig. 4B). Since the psychophysics test is presumed to reflect cortical GABAergic function, we subdivided the epilepsy cohort into two groups according to whether or not they were on drugs that are known to interact with GABA (Table 3; note that both groups contain people on polypharmacy). Notably, there was no difference in the SSI for these two groups (non‐GABA drug group, *n* = 27, SSI = 0.40 ± 0.37; GABA group, *n* = 27, SSI = 0.49 ± 0.36). Furthermore, including the presence or absence of drugs with GABAergic effects as a predictor in the regression analyses did not explain any additional variance (adjusted *R* ^2^ = 0.316). This was also true when the regression analyses were restricted to the epilepsy subjects (age/epilepsy subtype, adjusted *R* ^2^ = 0.475; age/epilepsy subtype/GABA effect, adjusted *R* ^2^ = 0.464). Finally, we examined whether patients with low versus high SSI scores (subdivided at the median SSI) were predominantly within the GABAergic/non‐GABAergic drug interactions groups (Fig. 4C). There was no significant difference between the low and high SSI patients (Fisher's exact tests), either for all the patients pooled irrespective of seizure type or for the generalized and focal groups alone. We concluded, therefore, that drug interactions do not underlie the effects of seizure type and age on the SSI.

**Table 3:** Subdivision of the drugs into those that are known to affect the GABAergic system, and those that are thought to have their effect independent of GABA

No documented GABA effect	
CBZ	Carbamazepine
ESL	Eslicarbazepine
LCM	Lacusamide
LEV	Leveliracetam
LTG	Lamolrigine
OXC	Oxcarbazepme
PER	Perampanel
PGB	Pregabalin
PHT	Phenyloin
Known GABAergic interactions	
CLB	Clobazam
MDZ	Midazolam
PB	Phenobarbital
PRM	Primidone
RTG	Retigabine
TPM	Topiramatc
VPA	Valproic acid
ZNS	Zonisamide

## Discussion

### SSI may provide biomarkers of epilepsy

The results of most general interest are the relationship between SSI with respect to the likelihood of seizure generalization, and the lack of a relationship with seizure frequency, especially since these are counter to what might have been anticipated. Our original hypothesis had been that people with epilepsy would have a reduced SSI, indicative of lowered inhibitory restraint. Instead, we found that as a group, patients with generalized seizures are no different from control subjects, but those with focal epilepsy that does not generalize (F^−^), have a raised SSI. This surprising finding contrasts with the reduced SSI in other groups: people with schizophrenia (Tadin et al. 2006a), depression (Golomb et al. 2009), low IQ (Melnick et al. 2013), and aged subjects (Betts et al. 2005; Yazdani et al. 2015). Notably, most of the previously noted associations with increased SSI are “good” factors (youth [Betts et al. 2005; Yazdani et al. 2015] and high IQ [Melnick et al. 2013]). The significantly raised SSI in the F^−^ patient group, relative to the other epilepsy groups, could not be explained by differences in age or IQ (there was no difference in ACE scores between the epilepsy groups). And while we cannot fully discount a confounding effect of concurrent depression, this condition is not known to be differentially associated with the presence, or absence, of generalized seizures in patients with focal epilepsy.

There are parallels between our study and a previous study of patients with migraine, who also showed evidence of increased suppression in a closely related perceptual task measuring contrast perception (Battista et al. 2011). The intriguing possibility is that in these patients with focal (nongeneralizing) epilepsy, the pathological activity is kept focused by an enhanced inhibitory restraint. Furthermore, it may therefore be possible to assess the quality of this restraint in regions of the cortex far removed from the focal pathology, as we do here with an assay of visual cortical function that appears to have relevance to foci elsewhere in the cortex. This presents an interesting question concerning whether the enhanced surround inhibition is independent of the epilepsy, or has arisen in reaction to the pathology, which will be addressed in future studies requiring longitudinal, repeated testing of patients from the time of diagnosis.

A large body of evidence has linked suppression of motion perception to processing in the motion area of visual cortex (cortical area MT) (Tadin et al. 2006b, 2003), equivalent approximately to the border between Brodmann areas 19 and 37 (see Tadin 2015 for an extensive review of this literature, including discussion of the involvement also of other parts of the visual system). With one exception (Ep12), this cortical area was not considered to be the focus of pathology for any of our patients (Table 1), which begs the question then of why measuring inhibitory function in a specific location may be relevant to epilepsy with a focus in a different part of the brain. There are parallels here with previous studies showing how SSI correlates with the occurrence of other brain pathologies not necessarily linked to visual processing, including schizophrenia (Tadin et al. 2006a) and depression (Golomb et al. 2009). Another study also showed an increase in contrast suppression in patients with migraine (Battista et al. 2011). We speculate that the answer lies in how inhibition may be affected globally, for instance, arising during development, or reflecting certain brain states, or under the influence of neuromodulators. If this is so, then an assay of inhibitory function at a particular location may also reflect inhibition in other areas that are relevant to the pathological condition.

The seizure frequency data are also interesting, although it needs to be interpreted with some caution, because this can be very difficult to estimate accurately (Hoppe et al. 2007). For instance, ambulatory recordings have shown that there is under‐reporting of many seizure events (Cook et al. 2013). With this caveat in mind, it is interesting to contrast the absence of any relationship between seizure frequency and SSI with that regarding the likelihood of seizure generalization: this difference suggests that seizure initiation and seizure generalization may occur through different mechanisms modulated by different factors.

For all groups, the association of SSI with age persisted, consistent with previous studies (Betts et al. 2005; Yazdani et al. 2015). It is noteworthy that the largest increases of SSI were found in young patients without a history of seizure generalization, and that this group showed a significantly steeper association. This may represent a progressive change in the risk of seizure generalization; undoubtedly some people in this group will at some stage in their life experience a generalized seizure, meaning that they would have moved epilepsy groups in our analysis. At an early age, then, these people might be considered “latent” with respect to seizure generalization. Furthermore, given the association between seizure generalization and sudden unexpected death in epilepsy (SUDEP), we speculate that having a relatively low SSI at the time of diagnosis, even without a history of seizure generalization, may be a poor prognostic indicator. Again, we will benefit from longitudinal studies of progression and variability in SSI in individuals with epilepsy.

Drug interactions were difficult to assess because the diverse drug regimes in our patient cohorts made it difficult to control for this variable. Since the SSI is considered to reflect cortical GABAergic interactions, we focused our attention on drugs that are known to modulate GABAergic activity. We performed several different analyses, showing that the different epilepsy cohorts had broadly similar pharma profiles, nor was there any apparent difference between patients with high and those with low SSIs. It remains a possibility that some drugs may interfere with performance on the test, but this is highly unlikely to explain the differences between the epilepsy groups.

### Usefulness for clinical practice

We have shown how a simple visual psychophysics test may provide a convenient and entirely noninvasive means of assessing the function of cortical networks in the clinical setting. These tests required only minimal training, and can provide a measure of SSI within 10 min. We adapted these to run on a tablet computer, thus providing a portable means of testing, which the patients could use either in the clinic, or in their own home. Importantly, the data collected on these tablet computers in the community and at clinics matched previous studies performed in laboratory conditions, in showing a progressive and highly significant decline in the SSI with increasing age.

The main clinical implication of our study relates to the association of SUDEP with generalized seizures. SUDEP affects approximately 1 in 1000 patients with epilepsy per year, and the single biggest risk factor is the presence of uncontrolled generalized tonic‐clonic seizures, increasing the risk to 1 in 150 patients per year (Nashef et al. 2007; Duncan and Brodie 2011; Shorvon and Tomson 2011). Currently, there are no reliable biomarkers of SUDEP risk. Any biomarker that reliably predicted patients at risk of generalized seizures would therefore be hugely beneficial for risk stratification, counseling, and treatment strategy. To be useful, such a biomarker would ideally be present before the occurrence of a first generalized seizure. We suggest that the SSI may prove to be a promising candidate for such a biomarker: the raised SSI seen in patients who have never previously had a generalized seizure indicating a lower risk of SUDEP, whereas the normal SSI seen in patients with a history of generalized seizures indicating a higher risk. Since SSI also tends to decrease with age, this index will be most useful for patients who develop, or are diagnosed with epilepsy early in life.

Our groupings according to seizure types were based on seizures that had already occurred, and we studied patients at only a single time point. We therefore cannot know whether the patients with generalized seizures had a normal SSI initially, and nor can we know whether patients with an increased SSI will remain free of generalized seizures in the long term. It is noteworthy, however, that the decline of SSI with age is significantly more steep in the F^−^ group than for other groups, which may mean that their risk of generalizing seizures, and therefore by extension, of SUDEP, may also change. These questions can only be addressed by further longitudinal studies. Nevertheless, given that visual psychophysical measures are simple, quick, and safe to administer, we feel that these further studies are justified.

## Conflict of Interest

None declared.

## References

[phy213079-bib-0001] Atallah, B. V. , W. Bruns , M. Carandini , and M. Scanziani . 2012 Parvalbumin‐expressing interneurons linearly transform cortical responses to visual stimuli. Neuron 73:159–170. 22243754 10.1016/j.neuron.2011.12.013 PMC3743079

[phy213079-bib-0002] Barlow, H.B. , and J. D. Mollon . 1982 The Senses. Cambridge University Press, Cambridge, U.K.

[phy213079-bib-0003] Battista, J. , D. R. Badcock , and A. M. McKendrick . 2011 Migraine increases centre‐surround suppression for drifting visual stimuli. PLoS ONE 6:e18211. 21494594 10.1371/journal.pone.0018211 PMC3073931

[phy213079-bib-0004] Berg, A. T. , S. F. Berkovic , M. J. Brodie , J. Buchhalter , J. H. Cross , W. van Emde Boas , et al. 2010 Revised terminology and concepts for organization of seizures and epilepsies: report of the ILAE Commission on Classification and Terminology, 2005‐2009. Epilepsia 51:676–685. 20196795 10.1111/j.1528-1167.2010.02522.x

[phy213079-bib-0005] Betts, L. R. , C. P. Taylor , A. B. Sekuler , and P. J. Bennett . 2005 Aging reduces center‐surround antagonism in visual motion processing. Neuron 45:361–366. 15694323 10.1016/j.neuron.2004.12.041

[phy213079-bib-0006] Betts, L. R. , A. B. Sekuler , and P. J. Bennett . 2009 Spatial characteristics of center‐surround antagonism in younger and older adults. J. Vis. 9:15–21. 10.1167/9.1.25 19271895

[phy213079-bib-0007] Cook, M. J. , T. J. O'Brien , S. F. Berkovic , M. Murphy , A. Morokoff , G. Fabinyi , et al. 2013 Prediction of seizure likelihood with a long‐term, implanted seizure advisory system in patients with drug‐resistant epilepsy: a first‐in‐man study. Lancet Neurol. 12:563–571. 23642342 10.1016/S1474-4422(13)70075-9

[phy213079-bib-0008] Duncan, S. , and M. J. Brodie . 2011 Sudden unexpected death in epilepsy. Epilepsy Behav. 21:344–351. 21665551 10.1016/j.yebeh.2011.04.056

[phy213079-bib-0009] Golomb, J. D. , J. R. McDavitt , B. M. Ruf , J. I. Chen , A. Saricicek , K. H. Maloney , et al. 2009 Enhanced visual motion perception in major depressive disorder. J. Neurosci. 29:9072–9077. 19605644 10.1523/JNEUROSCI.1003-09.2009 PMC2772577

[phy213079-bib-0010] Hoppe, C. , A. Poepel , and C. E. Elger . 2007 Epilepsy: accuracy of patient seizure counts. Arch. Neurol. 64:1595–1599. 17998441 10.1001/archneur.64.11.1595

[phy213079-bib-0011] Kleiner, M. , D. Brainard , D. G. Pelli , A. Ingling , R. Murray , and C. Broussard . 2007 What's new in Psychtoolbox‐3. Perception 36:1–16.

[phy213079-bib-0012] Melnick, M. D. , B. R. Harrison , S. Park , L. Bennetto , and D. Tadin . 2013 A strong interactive link between sensory discriminations and intelligence. Current Biol. 23:1013–1017. 10.1016/j.cub.2013.04.053 PMC3702042 23707433

[phy213079-bib-0013] Nashef, L. , N. Hindocha , and A. Makoff . 2007 Risk factors in sudden death in epilepsy (SUDEP): the quest for mechanisms. Epilepsia 48:859–871. 17433051 10.1111/j.1528-1167.2007.01082.x

[phy213079-bib-0014] Pouille, F. , O. Watkinson , M. Scanziani , and A. J. Trevelyan . 2013 The contribution of synaptic location to inhibitory gain control in pyramidal cells. Physiol. Rep. 1:e00067. 24303159 10.1002/phy2.67 PMC3841021

[phy213079-bib-0015] Prince, D. A. , and B. J. Wilder . 1967 Control mechanisms in cortical epileptogenic foci. “Surround” inhibition. Arch. Neurol. 16:194–202. 6018049 10.1001/archneur.1967.00470200082007

[phy213079-bib-0016] Read, J. C. , R. Georgiou , C. Brash , P. Yazdani , R. Whittaker , A. J. Trevelyan , et al. 2015 Moderate acute alcohol intoxication has minimal effect on surround suppression measured with a motion direction discrimination task. J. Vis. 15:15. 10.1167/15.1.5 25583875

[phy213079-bib-0017] Sengpiel, F. , R. J. Baddeley , T. C. Freeman , R. Harrad , and C. Blakemore . 1998 Different mechanisms underlie three inhibitory phenomena in cat area 17. Vision. Res. 38:2067–2080. 9797967 10.1016/s0042-6989(97)00413-6

[phy213079-bib-0018] Serrano‐Pedraza, I. , V. Romero‐Ferreiro , J. C. Read , T. Dieguez‐Risco , A. Bagney , M. Caballero‐Gonzalez , et al. 2014 Reduced visual surround suppression in schizophrenia shown by measuring contrast detection thresholds. Front. Psychol. 5:1431. 25540631 10.3389/fpsyg.2014.01431 PMC4261701

[phy213079-bib-0019] Shorvon, S. , and T. Tomson . 2011 Sudden unexpected death in epilepsy. Lancet 378:2028–2038. 21737136 10.1016/S0140-6736(11)60176-1

[phy213079-bib-0020] Tadin, D. 2015 Suppressive mechanisms in visual motion processing: From perception to intelligence. Vision. Res. 115:58–70. 26299386 10.1016/j.visres.2015.08.005 PMC4587336

[phy213079-bib-0021] Tadin, D. , J. S. Lappin , L. A. Gilroy , and R. Blake . 2003 Perceptual consequences of centre‐surround antagonism in visual motion processing. Nature 424:312–315. 12867982 10.1038/nature01800

[phy213079-bib-0022] Tadin, D. , J. Kim , M. L. Doop , C. Gibson , J. S. Lappin , R. Blake , et al. 2006a Weakened center‐surround interactions in visual motion processing in schizophrenia. J. Neurosci. 26:11403–11412. 17079669 10.1523/JNEUROSCI.2592-06.2006 PMC6674537

[phy213079-bib-0023] Tadin, D. , J. S. Lappin , and R. Blake . 2006b Fine temporal properties of center‐surround interactions in motion revealed by reverse correlation. J. Neurosci. 26:2614–2622. 16525040 10.1523/JNEUROSCI.4253-05.2006 PMC6675169

[phy213079-bib-0024] Trevelyan, A. J. , and C. A. Schevon . 2013 How inhibition influences seizure propagation. Neuropharmacology 69:45–54. 22722026 10.1016/j.neuropharm.2012.06.015

[phy213079-bib-0025] Trevelyan, A. J. , D. Sussillo , B. O. Watson , and R. Yuste . 2006 Modular propagation of epileptiform activity: evidence for an inhibitory veto in neocortex. J. Neurosci. 26:12447–12455. 17135406 10.1523/JNEUROSCI.2787-06.2006 PMC6674895

[phy213079-bib-0026] Walker, M. C. , and R. Surges . 2009 Mechanisms of antiepileptic drug action Pp. 75–91 *in* Shorvon S. D., Perucca E., Engel J., eds. The Treatment of Epilepsy. Wiley‐Blackwell, Hoboken, NJ.

[phy213079-bib-0027] Watson, A. B. , and D. G. Pelli . 1983 QUEST: a Bayesian adaptive psychometric method. Percept. Psychophys. 33:113–120. 6844102 10.3758/bf03202828

[phy213079-bib-0028] Wilson, N. R. , C. A. Runyan , F. L. Wang , and M. Sur . 2012 Division and subtraction by distinct cortical inhibitory networks in vivo. Nature 488:343–348. 22878717 10.1038/nature11347 PMC3653570

[phy213079-bib-0029] Yazdani, P. , I. Serrano‐Pedraza , R. G. Whittaker , A. Trevelyan , and J. C. Read . 2015 Two common psychophysical measures of surround suppression reflect independent neuronal mechanisms. J. Vis. 15:21. 10.1167/15.13.21 26401628

